# Supplementary material for: Applying the Behaviour Change Wheel to UK Local Authority Policy Documents: A Content Analysis in the Context of Financial Behaviour
Source: Behav Sci (Basel). 2023 Nov 30;13(12):991. doi: 10.3390/bs13120991 (PMC10741065; doi:10.3390/bs13120991)
Supplement: Supplementary file 1 [file behavsci-13-00991-s001.zip › behavsci-2683242-supplementary.pdf]

Appendix 1

Figure S1: Search strategy

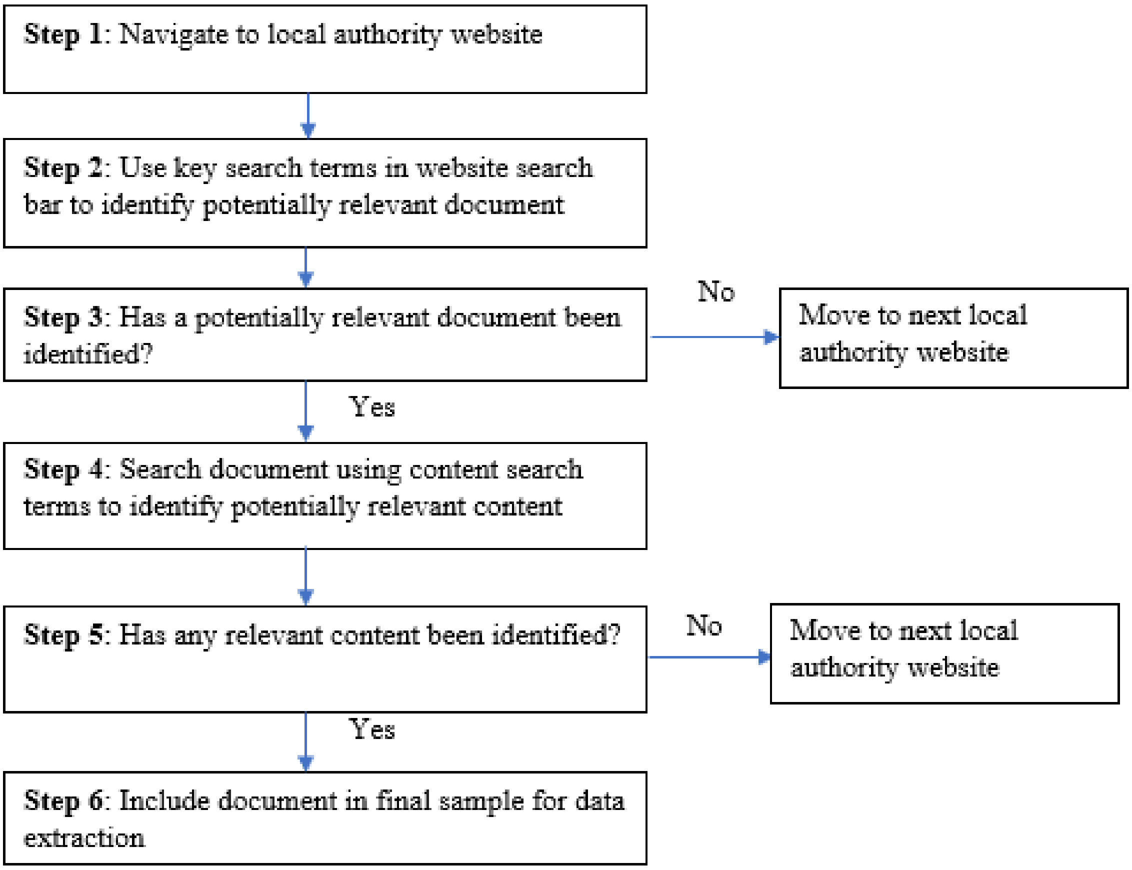

Appendix 2

Table S1: A sample of the coding form used and explanations for each criteria

LA	Extract code	Data fragment	Behavioural area of interest	RQ1: Has a target behaviour been specified?	RQ1: If yes - what is the target behaviour?	RQ1: Action	RQ1: Actor	RQ1: Context	RQ1: Target	RQ1: Time
Name of LA that published document	*Code for each extract*	*Every data fragment that was extracted for that document*	*Which behavioural area of interest is the data related to?*	*Yes/No*	*Free text box to aid the assessment of the target behaviour*	*Yes/No*	*Yes/No*	*Yes/No/MIN*	*Yes/No/MIN/NN*	*Yes/No/MIN*
Blackpool	BPL1	Encourage low income residents who are claiming either Working Tax Credit, Child Tax Credit or Universal Credit, to save by promoting the Help to Save savings account	Saving	Yes	resident saving	No	Yes	No	NN	No

LA	RQ2a: Has an intervention been specified?	RQ2a: If yes - what is the intervention?	RQ2a: Is the intervention able to be categorised using IFs?	RQ2a: Intervention type 1	RQ2a: Intervention type 2	RQ2a: Intervention type 3	RQ2a: Is the intervention able to be categorised using BCTTv1?	RQ2a: BCT 1	RQ2a: BCT 2	RQ2a: BCT 3	RQ2a: BCT 4	RQ2a: BCT 5
Name of LA that published document	*Yes/No*	*Free text box to aid the assesssment of the intervention*	*Yes/No*	*Drop down list of the nine intervention types*	*Drop down list of the nine intervention types*	*Drop down list of the nine intervention types*	*Yes/No*	*Drop down list of the 93 BCTs*	*Drop down list of the 93 BCTs*	*Drop down list of the 93 BCTs*	*Drop down list of the 93 BCTs*	*Drop down list of the 93 BCTs*
Blackpool	Yes	promoting the Help to Save savings account	Yes	Enablement	Education		No					

LA	RQ2b: Have any barries/enablers been specified?	RQ2b: If yes what are the barriers/enablers?	RQ2b: Are the barries/enablers able to be catergoriesed under COM-B?	RQ2b: COM-B component 1	RQ2b: COM-B component 2	RQ2b: COM-B component 3
Name of LA that published document	*Yes/No*	*Free text box to aid the assessment of the barriers/facilitators*	*Yes/No*	*Drop down list of the 6 COM-B components*	*Drop down list of the 6 COM-B components*	*Drop down list of the 6 COM-B components*
Blackpool	No					

Appendix 3

Table S2: Assessment of the common Action words within the documents

Action word	Relevant dictionary definition (Oxford English Dictionary, 2022)	Assessment of discrete	Assessment of observable
Save	To keep and store up instead of spending or using, esp. over time; (chiefly) spec. to accumulate (money) by regularly setting aside a portion of one's income, now typically by depositing sums in a bank or building society account, purchasing savings certificates, etc.	Possibly – this would depend if there was any additional information given on how and what the person might be saving.	Yes – this could be an observable Action in the context of saving money.
Claim	To demand as one's own or one's due; to seek or ask for on the ground of right.<br><br>To make a claim for (indemnity), esp. upon an insurance company.<br><br>To assert and demand recognition of (an alleged right, title, possession, attribute, acquirement, or the like); to assert as one's own, to affirm one's possession of.	No – not discrete as likely to take multiple successive Actions<br>(all definitions)	Possibly- could be observable but depends on the specific Action or outcome<br>(all definitions)
Complete	To bring to an end, finish (an Action, performance, work, a distance, period of time, etc.).	No – not discrete as likely to take multiple successive Actions.	No – Unlikely to be observable.
Apply	To make an approach to (a person) for information or aid; to have recourse or make application to, to appeal to; to make a (formal) request for.<br><br>To put oneself forward formally as a candidate for a position, esp. in a recruitment or selection process; to submit an application to do something, for a job or other position, or to an institution.	No – not discrete as likely to take multiple successive Actions.<br>(all definitions)	Possibly – could be observable but depends on the specific Action or outcome.<br>(all definitions)

Action word	Relevant dictionary definition (Oxford English Dictionary, 2022)	Assessment of discrete	Assessment of observable
Search	To look for or seek (someone or something); to make efforts to find or discover.<br><br>An act or instance of searching for information, images, etc., on a digital database or the internet, esp. by entering a particular string of words or characters into a search engine. Also: the systematic retrieval of information of this kind; the facility for this.	Possibly – Could be discrete (e.g. searching on an online search engine) but would depend if enough information was given to how and what the searches would be carried out.<br>(all definitions)	Possibly – Could be observable but would depend on the specific Action or outcome.<br>(all definitions)
Take up	The number of people accepting or claiming something offered, esp. state benefits.	No – not discrete as likely to take multiple successive Actions.	No – unlikely to be observable.
Pay	To give, transfer, or hand over (money, or its equivalent) in return for goods or services, or in discharge of an obligation; to deliver (a sum or amount owed).<br><br>To give to (a person, organization, etc.) money that is due for goods received, a service done, a debt or obligation incurred, etc.; to remunerate.	Possibly – Could be discrete but would need more information on how and what is being paid.<br>(all definitions)	Possibly – this could be an observable Action.<br>(all definitions)
Request	To ask (a person), esp. in a polite or formal manner, to do something.<br><br>An instance of asking for something, esp. in a polite or formal manner; a petition or expression of wish; a document expressing such a wish; (also) the thing which is asked for.<br><br>The Action, on the part of a specified person, of asking for some favour, service, etc., from another; the expression of one's desire or wish directly addressed to the person or persons able to gratify it.	Possibly – Could be discrete but would need more information on how and what is being requested.<br><br>(all definitions)	Possibly – this could be an observable Action.<br><br>(all definitions)
Contact	To get into contact or in touch with (a person).	No – this is not discrete as it is likely to take multiple successive Actions.	Possibly – this could be an observable

Appendix 4

File S1: Instructions for coding Behaviour Change Techniques (BCTs)

Instructions

- Select the most appropriate BCTs from the BCT Taxonomy v1, (Michie et al. 2013).
- Only code BCTs that are applied to residents.
- Select the relevant BCTs from the drop down list within the Excel cell.

Hints and tips

- It is possible that when an intervention has been specified it will not be described in enough detail to code a BCT. Indicate "No" in the column "Is the intervention able to be categorised using BCTTv1?" if this is the case.
- If you coded an intervention under a particular IT then use this as a guide to narrow the search for potentially relevant BCTs. For example, if you coded an intervention as Education, start by looking at the information and feedback based BCTs.

Appendix 5

Table S3: A list of the themes of coding discrepancies, and individual examples, that were referred to the third coder

Theme	Data fragment	1st coder (ZM)	2nd coder (KW)	3rd coder (DD) comments	Outcome
When to code for persuasion?	"Promote the Council's Digital Equipment Recycling Scheme (Digital Blackpool) to encourage the uptake of loans for repurposed council laptops and portable routers"- Blackpool, Financial Inclusion Strategy	Education	Persuasion	Agree with the first coder. Not enough detail to code for Persuasion.	Kept the first coder's initial codes. No amendments to the codebook.
	"Develop an awareness campaign to build awareness about the importance of financial management" Blackpool, Financial Inclusion Strategy	Education,	Education, Persuasion	Agree with the first coder. Not enough detail to code for Persuasion.	As above
Environmental restructuring vs Enablement?	"Providing a dedicated Advice Hub in the Council's Customer Service Centre to provide space for organisations offering debt, benefits and financial advice to low income residents" – Cambridge, Anti-Poverty Strategy	Environmental restructuring, Education, Enablement	Environmental restructuring, Education	Questioned which part of the intervention description would go above and beyond education and environmental restructuring.	Removed code for enablement for this fragment and any other similar.

Theme	Data fragment	1st coder (ZM)	2nd coder (KW)	3rd coder (DD) comments	Outcome
	"Tenants will be offered help and advice on welfare benefits and assistance in completing their housing benefit / universal credit application" – Mansfield, Rent Arrears Policy	Education, Enablement	N/A [this fragment was not included in the reliability coding but was submitted to third coded as an example of a common issue]		Kept the enablement code for this fragment as the assistance with completing application forms is a clear sign of enablement and is not covered by the education
When to code for "4.1 Instructions on how to perform the behaviour" BCT?	"Work with training centres and local schools to provide financial awareness training and build financial management skills for when they leave education and enter employment" - Blackpool, Financial Inclusion Strategy	No BCT code	Instruction on how to perform the behaviour, Social Support (practical)	Agreed with the first coder, not enough detail to code a BCT.	Kept 1st coders code. No update to the codebook.
	"Deliver and promote digital skills training to residents, to teach residents how to: Search for discounts, Search for financial information and advice…." - Blackpool, Financial Inclusion Strategy	Instruction on how to perform the behaviour	Instruction on how to perform the behaviour, Social Support (practical)	Agreed that the Instructions on how to perform the behaviour BCT should be coded here	As above
When to code for Social Support (practical) BCT?	"Provide and promote debt and budgeting advice to help people avoid getting into problem debt in the first place and to assist those who are" - Blackpool, Financial Inclusion Strategy	Social support (practical)	No BCT code	Thought this fragment was more about informing than providing support.	Social Support (practical) code was removed for this fragment.

**Theme**	**Data fragment**	**1st coder (ZM)**	**2nd coder (KW)**	**3rd coder (DD) comments**	**Outcome**
	**“**Employing a Financial Inclusion Officer to support Council tenants and other low income residents in financial crisis to access financial support, benefits and budgeting and debt advice” - Cambridge, Anti-Poverty Strategy	Social Support (practical)	No BCT code	Agreed with the Social Support (practical) code.	Stuck with the first coder’s code. No updates to the code book.
Would a Physical capability barrier ever be relevant?	“People on low incomes are less likely to able to afford internet access, which can make it difficult to apply for Universal credit and other benefits, access cheaper utility deals,” - Cambridge, Anti-Poverty Strategy	Physical Opportunity, Physical Capability	Physical Opportunity	Physical capability is only relevant to musculoskeletal functioning and physical skill.	Removed physical capability code.

Appendix 6

Table S4: A list of all the documents included in the final sample

Local Authority	Name of document	Year/s
Blackpool	Financial Inclusion Strategy	2022-27
Bournemouth Christchurch and Poole	Debt Management Policy	2022
Cambridge	Anti-Poverty Strategy	2020-23
Dacorum	Housing Income Management Strategy	2018-22
Dartford	Rent Arrears Policy	2022
Daventry	Anti-Poverty Strategy	2022-25
East Devon	Poverty Reduction Strategy	2021-24
East Devon	Income Management Strategy	2022
East Riding of Yorkshire	Financial Inclusion Strategy	2019-23
Hinckley and Bosworth	Rent Arrears and Recovery Policy	2022
Maidstone	Financial Inclusion Strategy	2021-26
Mansfield	Rent Arrears Policy	n/a
Medway	Rent Arrears Recovery Policy	2020-22
Norwich	Non-Commercial Debt Policy	n/a
Plymouth	Child Poverty Action Plan	2019-2022
Runnymede	Financial Wellbeing Strategy	2022-2027
Thanet	Income Management Policy	n/a
Uttlesford	Rent Arrears Policy	n/a
Wealden	Financial Inclusion Strategy	2020-2023
Welwyn Hatfield	Rent Arrears Policy	n/a
West Lancashire	Financial Inclusion Strategy	2021-24
Wokingham	Anti-Poverty Strategy	2022-26

Appendix 7

Table S5: Data fragments that did and did not meet the extraction criteria

**Data fragment**	**Strategy**	**Does it meet extraction criteria for any RQ?**	**Rationale**
"Encourage low income residents who are claiming either Working Tax Credit, Child Tax Credit or Universal Credit, to save by promoting the Help to Save savings account"	Blackpool: Financial Inclusion Strategy	Yes - RQ1, RQ3/RQ4	This data meets all the criteria for RQ1 and RQ3/4 as it is describing a relevant behaviour that is the target of intervention and also describes the intervention.
"It is the responsibility of the tenant to pay their rent and charges on time even if they are in receipt of benefits."	East Devon: Income management strategy	Yes – RQ1	This data is describing a behaviour that is relevant to the behaviours of interest (paying bills).
"Provide and promote the tools necessary for residents to check and claim the full range of support available to them."	Maidstone: Financial Inclusion strategy	Yes – RQ1, RQ3/RQ4	The data is describing a behaviour ("residents to check.") that is relevant to a behaviour of interest and is the target of an intervention. It also describes the intervention "(Provide and promote the tools..")
"Improve training and support to help people develop budgeting skills to avoid increasing debt in the future."	Wokingham: Anti-Poverty Strategy	Yes – RQ3/RQ4	This data describes an intervention aiming to change the resident's behaviour. The intervention was judged to be linked to one of the behaviours of interest (paying bills). In this instance, budgeting skills are closely related to being able to pay bills on time.
"Where adults do not possess the basic skills of money management to help them avoid getting into debt or being misled on money matters."	East Riding of Yorkshire: Financial Inclusion Strategy	Yes – RQ2	This data was judged to be describing a barrier (lack of money management skills) that was relevant to one of the behavioural areas of interest (paying bills).

Data fragment	Strategy	Does it meet extraction criteria for any RQ?	Rationale
“Financial exclusion can also be experienced by households in other ways, for example, in the East Riding of Yorkshire, many residents live in rural areas that are not well served by services and facilities commonly associated with financial inclusion.”	East Riding of Yorkshire: Financial Inclusion Strategy	No	This data was judged to contain a behavioural barrier (residents living in rural areas). However, it was not considered specific enough to be associated with any of the behavioural areas of interest.
“Encourage residents to purchase necessary insurance by promoting the benefits of the different types of insurance e.g. home insurance, life insurance, income protection insurance etc”	Blackpool: Financial Inclusion Strategy	No	This data was judged as behaviourally relevant as it was describing a target behaviour of an intervention. However, the behaviour 'residents to purchase necessary insurance' was not judged to be associated with any of the three behavioural areas of interest.
“Whilst the majority of tenants pay their rent on time, it is important we understand the reasons why tenants fall into rent arrears and where possible help them to manage their money and prioritise”	Dacorum: Housing income management strategy	No	This data was deemed to be describing a behaviour ("tenants pay their rent on time") and was linked to a behavioural area of interest (paying bills on time). However, it was judged to be describing the general context in which tenants pay their rent rather than as a target behaviour of a behaviour change strategy.
"Emergency support can be provided to help with daily living costs, housing-related costs and household items."	East Riding of Yorkshire: Financial Inclusion Strategy	No	This data was judged to be describing an intervention but was not judged to be explicitly referring to a behaviour change intervention. There was also not a clear connection to any of the behavioural areas of interest.
“Improve digital access through the development of digital skills and confidence, whilst ensuring that digital products and services meet the needs of residents”	East Riding of Yorkshire: Financial Inclusion Strategy	No	This data was judged to be describing a behaviour change intervention. However, there was not a clear connection to one of the behavioural areas of interest.

Data fragment	Strategy	Does it meet extraction criteria for any RQ?	Rationale
"Continue to deliver the "Warmer Winter" project with First Days and Share."	Wokingham: Anti-Poverty Strategy	No	This data was judged to be describing an intervention. However, there was no further explanation given regarding the "Warmer Winter" project. Data that did not explicitly describe the intervention or project being delivered was not considered relevant and not extracted.
"Agree negotiated payments based on the tenants income and reasonable expenditure"	Hinckley and Bosworth: Rent arrears and Recovery Policy	No	This data was describing an intervention to recover rent arrears. Although closely linked to one of the behavioural areas of interest (paying bills on time), intervention aimed at the recovery arrears was not extracted. This is because the recovery of arrears and debt management behaviours was deemed to be contextual different to the three healthy financial behaviours of interest that could prevent situations of debt and arrears.
